# KLHL14 is a tumor suppressor downregulated in undifferentiated thyroid cancer

**DOI:** 10.1038/s41420-024-02063-7

**Published:** 2024-06-22

**Authors:** Matteo Esposito, Antonella Migliaccio, Sara Carmela Credendino, Rufina Maturi, Nella Prevete, Gabriella De Vita

**Affiliations:** 1 Dipartimento di Medicina Molecolare e Biotecnologie Mediche (DMMBM), Università degli Studi di Napoli Federico II, Via Pansini 5, 80131 Napoli, Italy; 2 Dipartimento di Scienze Mediche Traslazionali (DiSMeT), Università degli Studi di Napoli Federico II, Via Pansini 5, 80131 Napoli, Italy; 3 Istituto per l’Endocrinologia e l’Oncologia Sperimentale (IEOS) “G. Salvatore”, Consiglio Nazionale delle Ricerche (CNR), Via Pansini 5, 80131 Napoli, Italy

**Keywords:** Oncogenesis, Thyroid cancer

## Abstract

KLHL14 is a substrate-binding subunit of Cullin-RING ligase 3 ubiquitin ligase complex, highly enriched in thyroid since early embryonic development, together with its antisense RNA KLHL14-AS. We have previously demonstrated that Klhl14-AS is a competing endogenous RNA regulating several differentiation and survival factors in thyroid cancer, acting as tumor suppressor. Recently, also KLHL14 has been shown to function as tumor suppressor in diffuse large B-cell lymphoma and in malignant mesothelioma. Here we show that KLHL14 expression is strongly reduced in anaplastic thyroid cancer, the less differentiated and most aggressive type of thyroid neoplasia. Such reduction is reproduced in different in vivo and in vitro models of thyroid cancer, being invariably associated with loss of differentiation. When Klhl14 expression is rescued in thyroid transformed cells, it reduces the cell proliferation rate and increase the number of apoptotic cells. On the other side, Klhl14 loss of function in normal thyroid cells affects the expression of several regulatory as well as functional thyroid markers. All these findings suggest that KLHL14 could be considered as a novel tumor suppressor in thyroid cancer, by also revealing its physiological role in the maintenance of a fully differentiated and functional thyroid phenotype.

## Introduction

Kelch-like (KLHL) protein family members are regarded as substrate-binding subunits of Cullin-Ring Ligase 3 (CRL3) E3 ubiquitin ligase. KLHLs commonly contain a BTB/poxvirus and zinc finger (POZ) domain, a BACK domain and a Kelch domain with five to six kelch repeated motifs. In a CRL3 complex, the BTB/POZ domain binds Cullin 3, the Kelch domain is responsible for specific substrate recruitment and the BACK domain acts as a linker [1]. KLHL proteins are thus modulators of substrate protein abundance via ubiquitination-dependent proteasome degradation and regulators of localization of target proteins, and generally display tissue-restricted expression profiles [2, 3]. Among members of this large family, KLHL14 was identified in neurons as a protein interactor of Torsin 1 A, whose mutations, responsible for the early-onset primary dystonia (Dyt1), disrupt such interaction [4]. Mice lacking Klhl14 die shortly after birth, while heterozygous knockout mice show an impaired development of a specific subpopulation of B lymphocytes (B-1a), demonstrating the relevance of this gene in lymphocytic lineage differentiation [5]. More recently, KLHL14 was reported to be involved in different human cancers. It has been shown that KLHL14 is frequently inactivated by somatic mutation in Diffuse Large B-Cell Lymphoma, causing the improper activation of NF-kB pathway through impaired quality control of B-cell receptor [6], and it is found to play an anti-oncogenic role in malignant mesothelioma, where its expression as well as its subcellular localization depend on TGF-β/EMT signaling [7]. Conversely, KLHL14 was found upregulated in ovarian cancer and in endometrial carcinoma, specifically in patients with poor prognosis [8, 9]. We have previously shown that *Klhl14* and its natural antisense gene, named *Klhl14-AS*, are among the most enriched genes in developing mouse thyroid bud, with their expression being maintained from early thyroid specification to the adult thyroid gland [10]. Recently, we have demonstrated that the long noncoding RNA encoded by *Klhl14-AS* plays a tumor suppressor role in thyroid cancer by acting as a competing endogenous RNA of thyroid differentiation genes [11]. The possibility that also KLHL14, representing a marker of thyroid follicular cells since their commitment, could be involved in thyroid neoplastic transformation has not been explored yet.

Thyroid cancer (TC) is the most common endocrine neoplasm and accounts for 3.4% of yearly new cancer diagnosis [12, 13]. Thyroid malignancies can be classified in different histotypes based on morphology and differentiation. Differentiated thyroid cancers (DTC) represent the majority of TC, including papillary thyroid carcinoma (PTC) follicular thyroid carcinoma (FTC) and oncocytic carcinoma (OC), exhibiting various grades of differentiation. Poorly differentiated thyroid carcinoma (PDTC) and anaplastic thyroid carcinoma (ATC) are instead less frequent and with poorer prognosis than the differentiated histotypes [14]. Although the mechanisms of thyroid cancer progression are still debated, thyroid tumors most commonly feature mutations of *BRAF* and *RAS* that activate MAPK signaling with different strength, therefore producing a spectrum of tumor differentiation aberrations [13]. Constitutive activation of such signaling pathway sets off the loss of key thyroid-specific markers, that prevents cancer cells from being a functional tissue [15, 16]. Thyroid follicular cells express indeed a tissue-specific combination of transcription factors FoxE1, Pax8 and Titf1/Nkx2-1 [17–19] that are necessary for the expression of thyroid specific structural and functional markers such as thyroglobulin (Tg), sodium iodine symporter (NIS), thyroperoxidase (Tpo) and thyroid stimulating hormone receptor (Tshr). We have previously demonstrated that high levels of overexpression of exogenous *Ha-Ras* oncogene in FRTL-5 cells recapitulate reduction of thyroid markers, increased cell motility and loss of differentiation observed in poorly differentiated TC [20]. It is worth noting that differentiation state is particularly relevant in TC, as the responsiveness to radioiodine therapy is limited to differentiated NIS-expressing cells that retain the iodine uptake ability.

Here we show that KLHL14 is strongly downregulated in TC, particularly in the anaplastic histotype. We confirmed that such reduction is an early and common event of thyroid neoplastic transformation, either in vivo by using a BRAF^V600E^ mouse model of TC and in vitro in RAS oncogene-transformed rat thyroid follicular cells, where we also show that oncogenic activation directly represses the activity of the KLHL14 promoter. We then performed loss/gain of function experiments showing that Klhl14 rescue in RAS-transformed FRTL5-cells affects proliferation and increase cell death. Taken together our data indicate KLHL14 as a possible novel tumor suppressor gene in thyroid cancer.

## Results

### Klhl14 expression is decreased in undifferentiated thyroid cancer cells

**Fig. 1:**
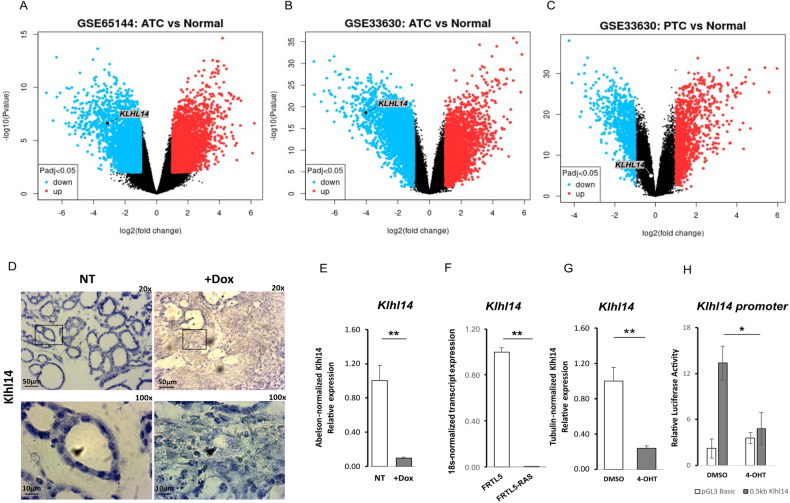
KLHL14 expression in human and experimental thyroid cancers. Volcano Plots depicting differential gene expression in anaplastic thyroid carcinomas (ATC) from gene expression dataset GSE65144 (**A**), GSE33630 (**B**) and in papillary thyroid carcinomas (PTC) from GSE33630 (**C**), in which *KLHL14* scattering is highlighted. The x-axis enlist the log2 fold change value representing the mean expression value of each gene, while the y-axis is the level of significance (adjusted p-value) reported in –log10; *blue dots* represent significantly down-regulated genes in the first group, *red dots* represent significantly up-regulated genes. **D** In situ hybridization micrograph showing *Klhl14* RNA-probe signals in normal thyroid of Tg-rtTA; TetO/BRAF^V600E^ untreated mouse (NT) and in neoplastic thyroid of doxycycline treated mouse (+ Dox), at two different magnifications. *Klhl14* mRNA levels analyzed through qRT-PCR in (**E**) thyroids from either untreated (NT) or treated (+Dox) Tg-rtTA; TetO/BRAF^V600E^ mice, (**F**) parental FRTL5 and transformed FRTL5-RAS cells, (**G**) FRTL5-ER^TM^-RAS cells either treated with vehicle (DMSO) or 4-hydroxytamoxifen (4-OHT). **H** Luciferase activity induced by Klhl14 promoter (0.5 kb Klhl14) and empty reporter vector (pGL3Basic) in FRTL5-ER^TM^-RAS cells either treated with vehicle (DMSO) or 4-hydroxytamoxifen (4-OHT). Relative luminescences were normalized on Renilla luciferase activity used as internal control. Statistical t-test significance is reported as: **, *p*-value < 0.01; *, p-value < 0.05; ns not statistically significant.

To investigate the possible involvement of Klhl14 in thyroid neoplastic transformation we compared its expression in samples of human anaplastic thyroid cancers (ATC), papillary thyroid cancers (PTC) and normal tissue, employing GEO2R software to explore two publicly available datasets [21, 22]. As it is shown in the volcano plots, *KLHL14* is significantly downregulated in ATC from both datasets (Fig. 1A, B), while in PTC its downregulation is not significant (Fig. 1C). These data were confirmed in Tg-rtTA; TetO/BRAF^V600E^ mice, a mouse model of doxycycline (Dox)-inducible thyroid cancer, that develops undifferentiated thyroid carcinoma after 1 week of Dox treatment [23]. Thyroids from treated and control mice were analyzed for Klhl14 mRNA expression both by in situ hybridization (ISH) and RT-PCR showing a severe decrease of *Klhl14* levels in induced cancers respect to that observed in the control samples (Fig. 1D, E). We then extended the analysis to an in vitro model of undifferentiated thyroid cancer based on FRTL5 rat thyroid cells transformed by oncogenic Ras expression, either chronic or inducible (FRTL5-Ras and FRTL5-ER^TM^-Ras, respectively) [19, 24]. FRTL5-Ras cells show a strong reduction of *Klhl14* expression respect to the parental FRTL5 cells (Fig. 1F). Consistently with the chronically transformed cells, also in FRTL5-ER^TM^-Ras cells *Klhl14* transcript expression dropped following acute Ras induction by tamoxifen (Fig. 1G). To investigate if Ras downregulates *Klhl14* expression at transcriptional level, we analyzed the effects of Ras activation on Klhl14 promoter activity using a minimal *Klhl14* promoter-driven luciferase reporter vector in FRTL5-ER^TM^-Ras cells [23]. Upon induction with tamoxifen, the promoter activity is greatly reduced compared to untreated FRTL5-ER^TM^-Ras cells (Fig. 1H). These findings show that KLHL14 expression is lost in human undifferentiated thyroid cancer as well as in different experimental models of such tumors, similarly to what happens to the well-established thyroid differentiation markers [13, 15].

### Klhl14 rescue impairs thyroid transformed cells growth

**Fig. 2:**
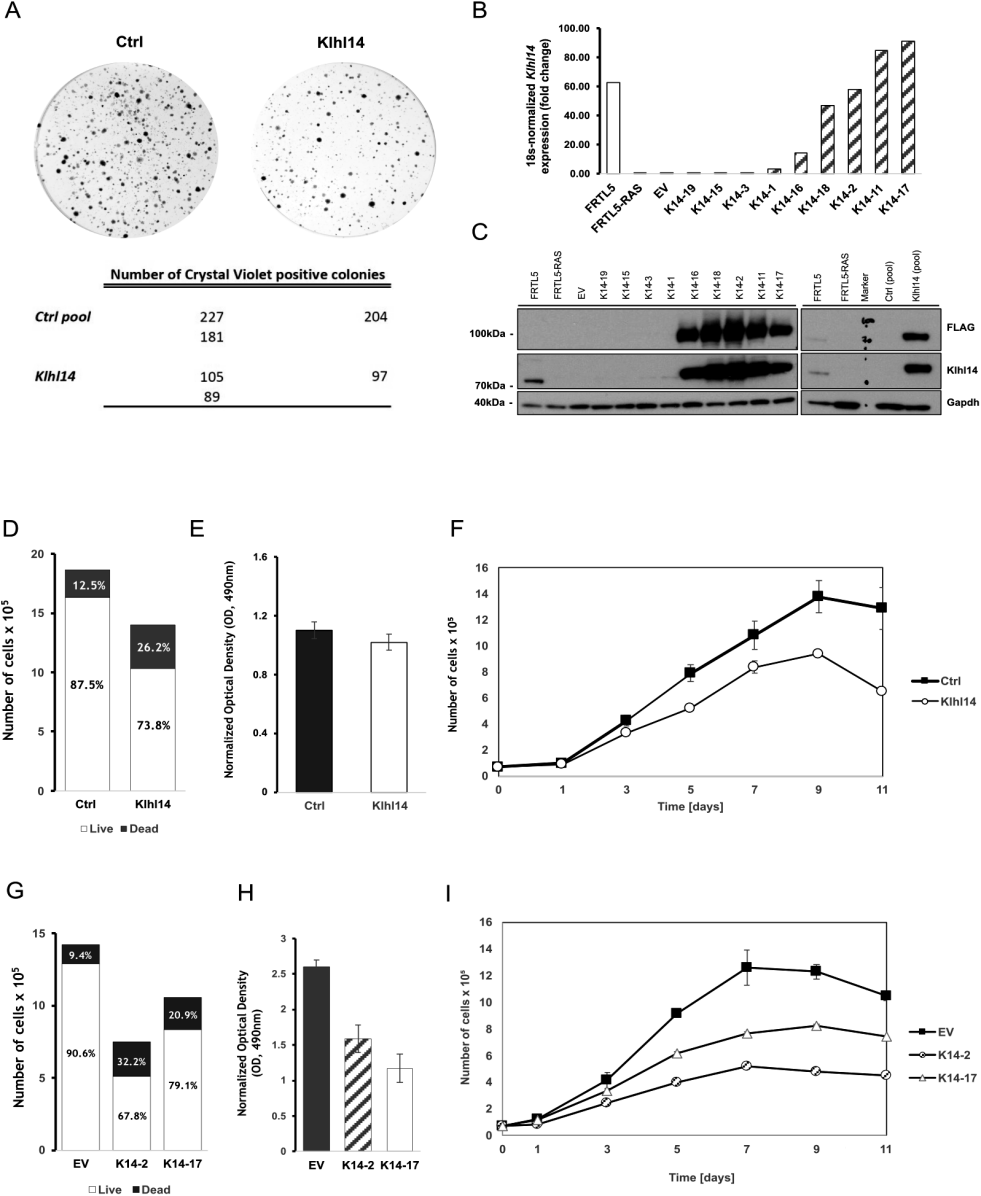
Klhl14 expression affects proliferation and viability of transformed thyroid cells. **A** Clonogenic assay of FRTL5-HRasG12V cells transfected with Klhl14 (Klhl14) or control plasmid (Ctrl), and their respective absolute and average colony numbers from two independent experiments. **B** Klhl14 expression measured by qRT-PCR in FRTL5, FRTL5-RAS, FRTL5-RAS control clone (EV) and in nine different FRTL5-RAS Klhl14-expressing clones. Clones were ordered based on ectopic Klhl14 expression levels, normalized on that of FRTL5-RAS cells. **C** Klhl14 expression measured by immunoblot in FRTL5, FRTL5-RAS, FRTL5-RAS control clone (EV) and in nine different FRTL5-RAS Klhl14-expressing clones, as well as in FRTL5-RAS control (Ctrl) and Klhl14 pools. Klhl14 was detected by both anti-FLAG antibody (upper panels) and anti-Klhl14 antibody (lower panels). Gapdh was used as loading control. Uncropped blots are in Supplementary Fig. 1. Cell viability of FRTL5-RAS Klhl14 and control pools assessed by trypan blue exclusion assay (**D**) and MTS assay (**E**). **F** growth curve of FRTL5-RAS Klhl14 and control pools. Cell numbers are reported as “cells x 10^5^”. Cell viability of control (EV) and two Klhl14-expressing FRTL5-RAS clones (K14-2 and K14-17) assessed by by trypan blue exclusion assay (**G**) and MTS assay (**H**). **I** Growth curve of control (EV) and two Klhl14-expressing FRTL5-RAS clones (K14-2 and K14-17). Cell numbers are reported as “cells x 10^5^”.

To establish if Klhl14 reduction plays a functional role in cancer, we tested if Klhl14 ectopic expression in thyroid neoplastic cells could interfere with the transformed phenotype. To this purpose, we used FRTL5-Ras, chronically transformed thyroid cells with no detectable endogenous Klhl14 expression (Fig. 1F) presenting high proliferation rates and a severe impairment in their differentiation status respect to the parental ones [16, 17]. Klhl14 expression was restored by transfecting a 3x-Flag-Klhl14 encoding vector, or a control empty vector, in FRTL5-Ras cells and generating both neomycin-resistant mass populations and several individual clones. Clonogenic assays performed in duplicate plates revealed that the number of colonies in Klhl14-transfected plates was significantly lower than that in control plates (97 vs. 204 colonies on average) (Fig. 2A) while simultaneously showing a smaller colony diameter compared to the controls. Klhl14-transfected individual clones (K14 clones) and mass population (K14 pool), together with an empty vector (EV)-transfected clone (EV clone) and mass population (EV pool) were analyzed by qRT-PCR for Klhl14 mRNA levels (Fig. 2B) and by immunoblot for Klhl14 protein abundance (Fig. 2C). Among the screened clones, K14-2 and K14-17 were selected for further analyses. Cell viability via Trypan Blue exclusion assay revealed that the K14 pool had a reduced percentage of live cells respect to the control (Fig. 2D). Overall cell viability assessed by MTS assay revealed a slight reduction in MTS oxidation rate in the K14 pool that could explain a possible metabolic impairment (Fig. 2E). Cell counting performed on growing cultures revealed that Klhl14-expressing cells tend to proliferate less and to reach plateau growth earlier than control cells, reinforcing the possibility that Klhl14 could suppress neoplastic cell growth (Fig. 2F). The same analyses performed in Klhl14-expressing clones confirmed the effects observed in the mass population. Indeed, both K14-2 and K14-17 clones had lower percentages of living cells compared to EV control clone (Fig. 2G). Metabolic activity in the two clones was about one half of that of the control clone (Fig. 2H). Growth curve analysis performed on K14 clones showed that the restoration of Klhl14 negatively affects proliferation and anticipates plateau phase of growth (Fig. 2I).

### Klhl14 induces an impairment in DNA synthesis and increases apoptosis in thyroid neoplastic cells

**Fig. 3:**
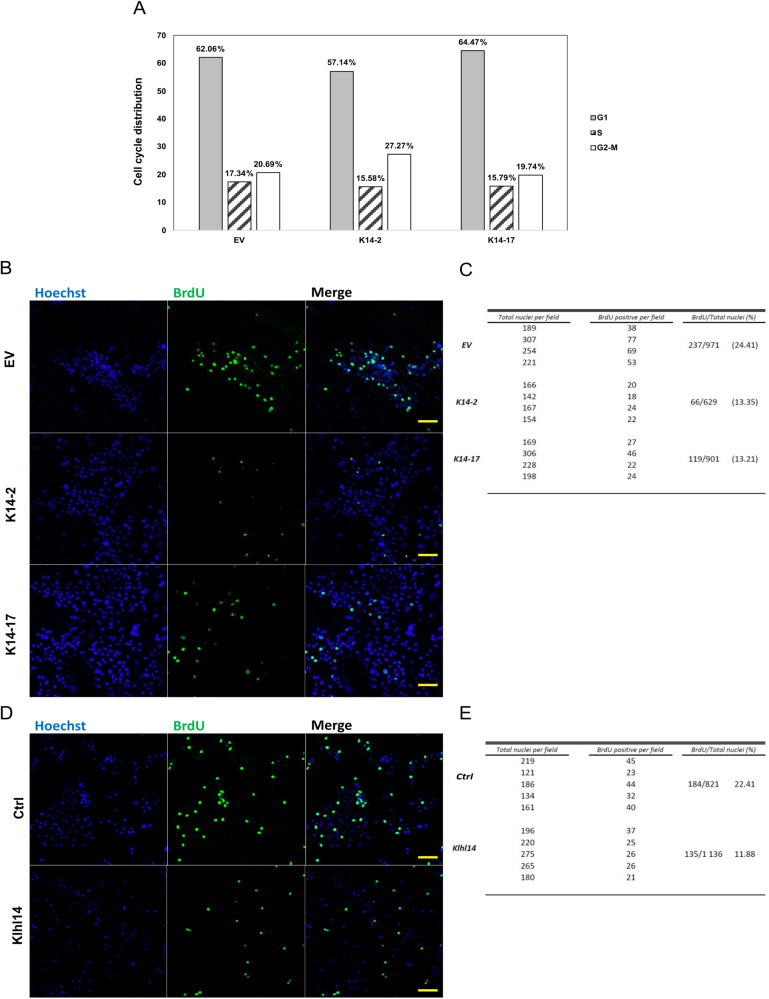
Klhl14 expression reduces S-phase entry of transformed thyroid cells. **A** Cell cycle analysis by flow cytometry using propidium iodide staining in two FRTL5-RAS Klhl14-expressing clones and one control clone (EV). The histograms report the percentage of cells in G1, S, and G2-M phases of the cell cycle. Fluorescence micrographs of BrdU incorporation assay in (**B**) FRTL5-RAS Klhl14-expressing and control (EV) clone and in (**D**) FRTL5-RAS Klhl14 expressing and control (Ctrl) mass populations, taken at 20x magnification. Cells from four acquired fields were counted for BrdU positivity and reported respectively in (**C**, **E**). BrdU assays shown are representative of two independent replicates. Scale bar (50 µm) is added for reference.

The above data on cell growth brought us to hypothesize that Klhl14 could take the role of tumor suppressor by impairing cell cycle and/or triggering apoptosis. To analyze cell cycle, we performed a Propidium Iodide Cell Sorting assay on non-synchronized K14 and EV clones. Notably, Klhl14-expressing clones exhibited a slight misdistribution of cells at the different cycle stages compared to the control clone. Indeed, both Klhl14 expressing clones displayed a reduced percentage of *S-phase* cells, with K14-2 also presenting a lower percentage of *G1-phase* cells and a higher percentage of *G2/M-phase* cells (Fig. 3A).

To confirm that Klhl14 impairs cell cycle of thyroid neoplastic cells, we performed BrdU proliferation assay, showing that Klhl14 expressing clones incorporated less BrdU respect to the control, with about 13% of BrdU-positive cells in both K14-2 and K14-17 clones versus 24.41% of BrdU positive cells in EV clone (Fig. 3B, C). The same experiment repeated on Klhl14 pool confirmed a lower duplication rate in Klhl14 compared to control pool (11.88% of BrdU positive cells in K14 pool vs. 22.41% of BrdU positive cells in EV pool) (Fig. 3D, E).

**Fig. 4:**
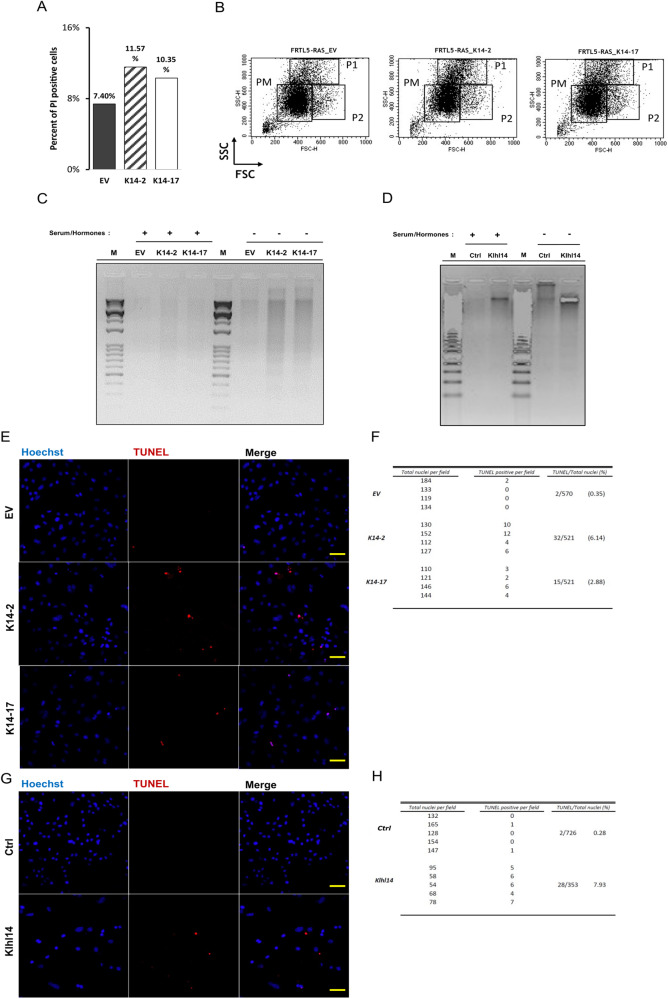
Klhl14 expression increases cell death in transformed thyroid cells. **A** Cytometric evaluation of cell viability using propidium iodide in non-permeabilized FRTL5-RAS Klhl14-expressing and control (EV) clones. Percentages of dead cells are reported. **B** Flow cytometry scatter plots of Klhl14 and EV clones. Size (forward scatter) and granularity (side scatter) On the x-axis are plotted the Forward Scattering intensity values (FSC-H), while on the y-axis there are the Side Scattering intensity values (SSC-H) relative to 10,000 events registered. Agarose gel electrophoresis of fragmented DNA from (**C**) FRTL5-RAS Klhl14-expressing (K14-2 and K14-17) and control (EV) clones and (**D**) FRTL5-RAS Klhl14-expressing (Klhl14) and control (Ctrl) mass populations, cultured either in the presence (+) or absence (−) of serum and hormones. Fluorescence micrographs of TUNEL assay in (**E**) two FRTL5-RAS Klhl14 expressing and onecontrol (EV) clones and (**G**) FRTL5-RAS Klhl14 expressing and control (Ctrl) mass populations, taken at 20x magnification. Cells from four acquired fields were counted for TUNEL positivity and reported, respectively, in (**F**, **H**). Presented results are representative of two independent replicates. Scale bar (50 µm) is added for reference.

We further characterized K14 clones by propidium staining of unpermeabilized cells, followed by cell sorting, observing a larger propidium iodide uptake by K14 clones compared to EV clones (Fig. 4A). This, together with the Trypan Blue exclusion assay shown in Fig. 2B, indicates higher mortality rate in Klhl14 clones. Flow cytometry assay of the same cells revealed an altered segregation of Klhl14 clones in three populations with different light-scattering characteristics. Beside the predominant population present in each clone and control (PM), two populations with higher value of either granularity (side scattering, P1) or volume (forward scattering, P2) are more densely crowded in Klhl14-expressing clones than in control (Fig. 4B).

These findings strongly suggest that Klhl14 restoration can lead cancer cells to apoptosis. As a matter of fact, subsequent DNA fragmentation analyses revealed that Klhl14 clones displayed a DNA fragmentation pattern typically associated to caspase associated DNase (CAD) activation, that became even more evident when Klhl14 clones were serum-starved for 72 h (Fig. 4C). The same analysis performed on Klhl14 expressing cell pools led to similar results, as the fragmentation pattern is evident in Klhl14 pools, while it is absent in control cell pool (Fig. 4D). To confirm Klhl14 involvement in enhancing susceptibility to apoptosis, TUNEL assay was performed on the two clones and on the pool. As shown in immunofluorescent micrographs and table in Fig. 4E, F, both K14 clones display an exacerbated DNA damage compared to EV control clone (6.14% TUNEL-positive cells in K14-2 and 2.88% of TUNEL-positive cells in K14-17, versus 0.35% TUNEL-positive cells in EV). Klhl14 cell pool confirmed the increase of DNA fragmentation rate (7.93% TUNEL-positive cells in K14 pool vs. 0.28% in control pool) (Fig. 4G, H).

### Modulation of Klhl14 alters the expression of key thyroid differentiation markers

**Fig. 5:**
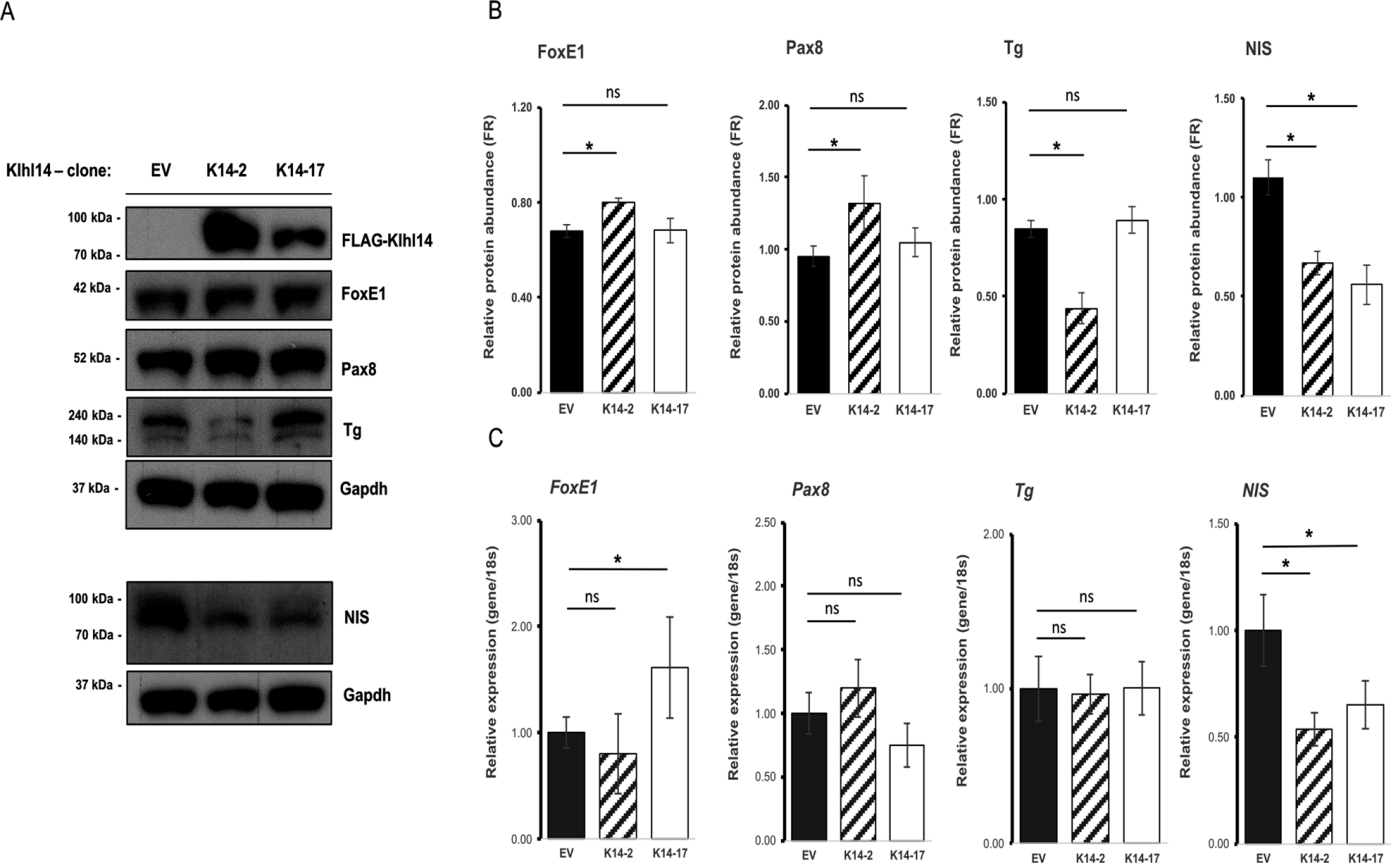
Thyroid differentiation markers expression in Klhl14 expressing thyroid transformed cells. **A** Immunoblots showing protein expression of flagged-Klhl14 and thyroid markers in two FRTL5-RAS (K14-2 and K14-17) and control (EV) clones. Gapdh was assayed for each series of markers analyzed on the same blot. **B** Immunoblot signal quantifications of thyroid marker proteins normalized on the respective Gapdh. **C** Thyroid markers mRNA levels assessed by qRT-PCR on the same clones as in (**A**). Data are represented as mean ± SD of two independent replicates. Statistical t-test significance is reported as: *, *p*-value < 0.05; ns not statistically significant. Uncropped blots are in Supplementary Figs. 2–4.

**Fig. 6:**
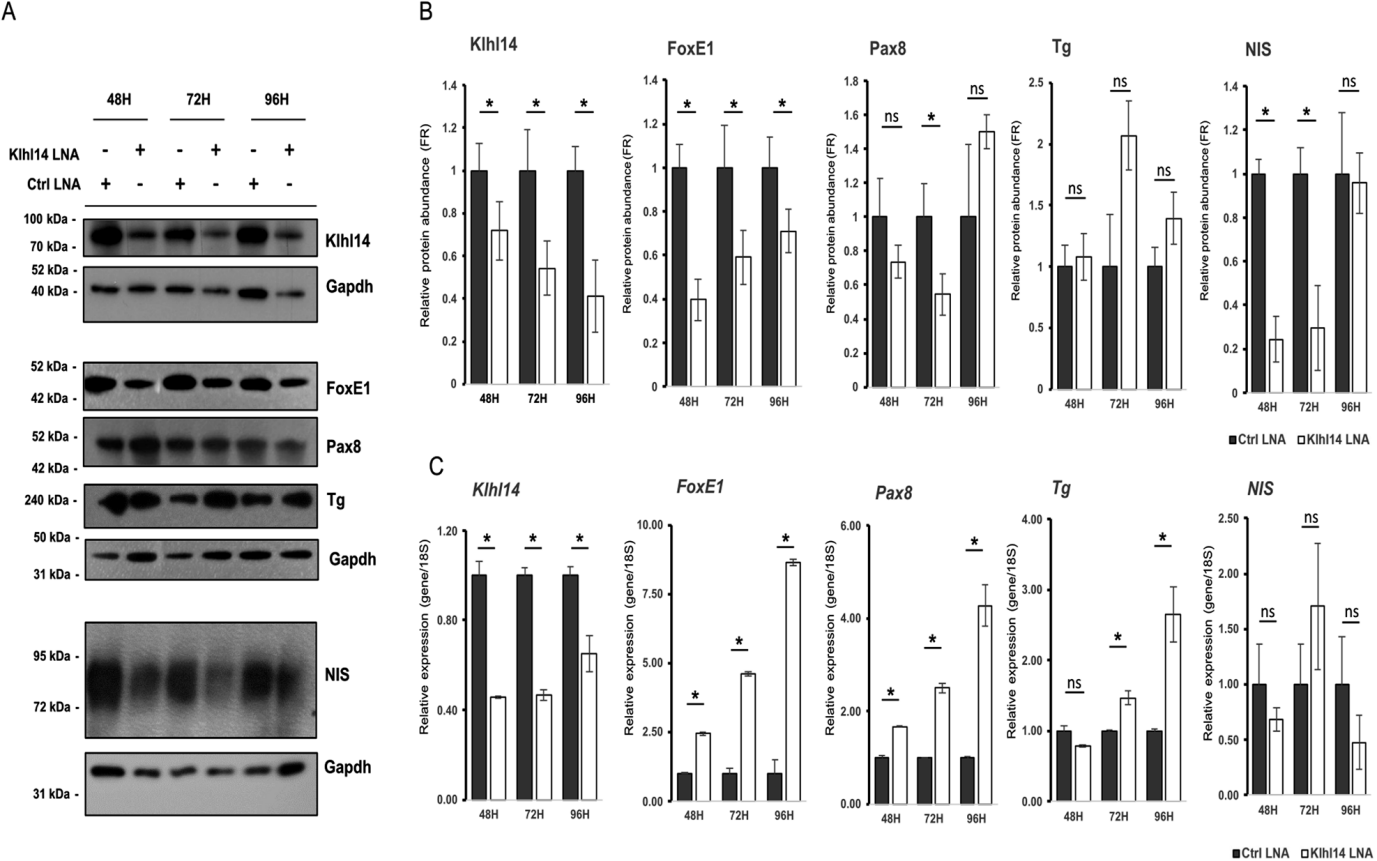
Effects of Klhl14 knockdown on normal thyroid differentiation. Klhl14 was knocked down in FRTL5 cells by a specific LNA (Klhl14 LNA) or scrambled LNA (Ctrl LNA) and analyzed at 48H, 72H and 96H post-transfection. **A** Immunoblots showing protein expression of endogenous Klhl14 and thyroid markers. Gapdh, for each immunoblot, is shown as loading control. **B** Immunoblot signal quantifications of thyroid marker proteins normalized on the respective Gapdh. **C** Thyroid markers and endogenous *Klhl14* mRNA levels assessed by qRT-PCR. Data are represented as mean ± SD of three independent experiments. Statistical t-test significance is reported as: *, *p*-value < 0.05; ns not statistically significant. Uncropped blots are in Supplementary Figs. 5−7.

Since Klhl14 reduction was specifically observed in human ATC, the less differentiated type of thyroid carcinoma, we asked whether Klhl14 restoration could change the differentiation status of transformed cells. Western blot analysis showed that the expression of Klhl14 protein was higher in K14-2 than K14-17 (Fig. 5A). Both FoxE1 and Pax8 significantly increased their expression only in K14-2 clone. Thyroglobulin decreased significantly in K14-2, while its abundance in K14-17 was comparable to that of control clone. NIS protein was the only marker whose expression was significantly decreased in both clones in respect to the control (Fig. 5B). We measured the transcript levels of the same markers, showing that Klhl14 restoration did not alter transcript levels of most studied markers, except for FoxE1, whose levels increase only in K14-17, and NIS, that was instead significantly lower in both K14-2 and K14-17 respect to control clone (Fig. 5C). Overall, these data show that Klhl14 restoration in chronically transformed thyroid cells affects expression levels of specific differentiation genes, with NIS protein and mRNA being consistently reduced in both examined clones. Such effects of Klhl14 expression in transformed thyroid cells prompted us to ask whether Klhl14 might play a role on thyroid differentiation status of normal thyroid cells. To investigate such role in thyroid, we knocked down Klhl14 expression in FRTL-5 normal rat thyroid follicular cells by RNA interference and analyzed the expression of specific differentiation markers at three time points of 48, 72 and 96 h. Klhl14 protein abundance was decreased to 0.75-fold over control at 48 h and to 0.4-fold over control at 96 h post-transfection (Fig. 6A, B). The protein levels of both FoxE1 and Pax8 were lower than the control at 48 (0.396 ± 0.095 *FR* and 0.735 ± 0.079 *FR*, respectively) and 72 h (0.591 ± 0.122 *FR* and 0.544 ± 0.116 *FR*, respectively) following Klhl14 knockdown, while only FoxE1 levels maintain a significant downregulation at 96 h (Fig. 6A, B). Thyroglobulin protein levels alterations are non-significant, while the protein abundance of NIS was strongly reduced respect to control samples (0.243 ± 0.105 *FR* and 0.296 ± 0.193 *FR* at 48 and 72 h respectively), although its levels returned to baseline at 96 h. The expression of the same markers was examined at the mRNA level (Fig. 6C). Klhl14 mRNA, as expected, was downregulated at all time points examined. Both *FoxE1* and *Pax8* mRNAs were instead significantly increased already at 24 h, with their levels continuing to grow at 72 and 96 h. *Tg* transcript was also upregulated upon Klhl14 interfering, but the increase was detected only at 72 and 96 h. *NIS* mRNA expression did not significantly vary over the time points. Interestingly, while *Klhl14* transcript decrease precedes that of its protein, the amount of *FoxE1* and *Pax8* transcripts steadily increased over the period of observation, suggesting the possibility that a feedback compensation mechanism could be triggered by the decrease in the corresponding proteins. These results suggest that Klhl14 could be considered an essential gene in the maintenance of normal thyroid differentiation.

### Nucleocytoplasmic distribution of Klhl14 is altered in thyroid neoplastic cells

**Fig. 7:**
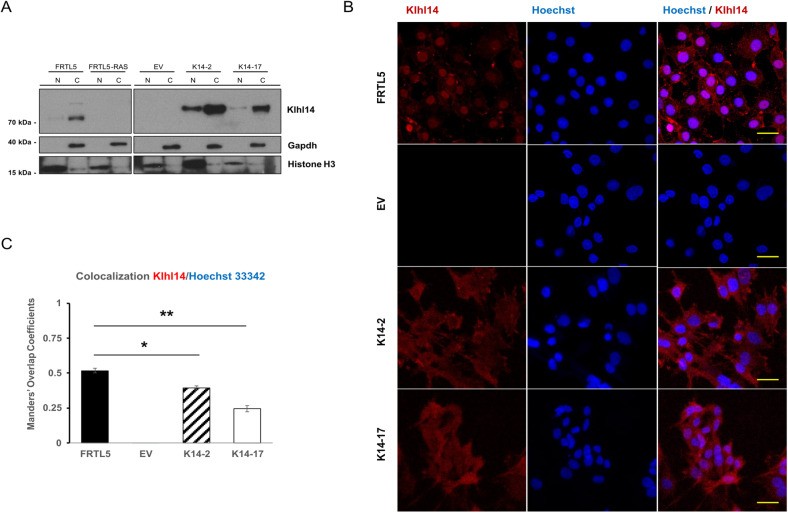
Klhl14 localization in normal and transformed thyroid cells. **A** Immunoblot showing the expression of Klhl14 protein in nuclear and cytoplasmic fractions, in FRTL5 cells, FRTL5-RAS cells, FRTL5-RAS clones (K14-2 and K14-17) and control EV. Klhl14 was detected by anti-Klhl14 antibody. Gapdh and Histone H3 are shown as loading controls of cytoplasm and nucleus, respectively. Signals from either endogenous or ectopic Klhl14 were captured at different exposure times to avoid signal saturation in overexpressing clones. Uncropped blots are in Supplementary Fig. 8. **B** Immunofluorescence micrographs of Klhl14 in FRTL5 cells and FRTL5-RAS clones (K14-2 and K14-17) and control EV, taken at 63x magnification (oil immersion objective). Scale bar (20 µm) is added for reference. Images from either endogenous or ectopic Klhl14 were captured at different exposure times to avoid signal saturation in overexpressing clones. **C** Manders’ Overlap Coefficients of FRTL5 cells and FRTL5-RAS clones (K14-2 and K14-17) and control EV, calculated as overlapping signals of Klhl14 channel in Hoechst 33324 channel. Pearson Colocalization Coefficients: FRTL5 *r* = *0.518*, EV *r=not applicable*, K14-2 *r* = *0.349*, K14-17 *r* = *0.245*. The *p*-value for this analysis was 100%, indicating a high probability of colocalization.

It has recently been shown that KLHL14 anti-oncogenic action in malignant mesothelioma is linked to changes in its nuclear-cytoplasmic shuttling in response to oncogenic stimuli [7]. For this reason, we asked how Klhl14 was distributed in thyroid cells and whether neoplastic transformation could alter its subcellular localization. To answer this question Klhl14 localization in parental FRTL5 was compared to that in transformed K14 clones. Immunoblot on nucleus/cytoplasm fractionated cell lysates showed that endogenous Klhl14 is prevalently localized in cytoplasm, although it is also detectable in nuclear fraction of FRTL5. Likewise, overexpressed flagged-Klhl14 in K14-2 and K14-17 was detected more strongly in the cytoplasm than in the nucleus. As expected from previous data (Fig. 2C), Klhl14 is not expressed in both FRTL5-Ras and EV clone used as controls (Fig. 7A). Immunofluorescence confirmed that Klhl14 is present in both the nucleus and cytoplasm of thyroid cells, revealing also a difference in nuclear enrichment of the signal, which appears to be greater in parental FRTL5 compared to transformed clones (Fig. 7B). The difference was confirmed by quantification of colocalization of Klhl14 and DAPI signals, showing a lower overlap coefficient in clones K14-2 and K14-17 than that in FRTL5 cells (Fig. 7C).

## Discussion

In this paper we show that the E3 ubiquitin ligase component KLHL14 is strongly reduced in human and in vivo experimental undifferentiated thyroid cancer and is able to reduce proliferation and survival in thyroid neoplastic cells in vitro. We also demonstrate that Klhl14 is essential in normal thyroid cells for the maintenance of a differentiated phenotype in vitro. The interest in Klhl14 stems from previous observations showing that, together with its antisense transcript Klhl14-AS, it is among the most enriched genes in thyroid since early embryonic development, together with the already known differentiation markers [25]. We have already demonstrated that the noncoding Klhl14-AS regulates thyroid differentiation and survival by acting as ceRNA in a network including Pax8 and Bcl2 [11]. Here, by analyzing Klhl14 expression in papillary and anaplastic thyroid cancers collected in two publicly available datasets, we reveal that this gene behaves like the well-established thyroid differentiation markers, being strongly and significantly reduced in ATC, while its reduction is less evident and non-significant in PTC, similarly to what we already observed for its antisense [26]. This observation was confirmed in experimental models, both in vivo in a mouse model of Braf-driven thyroid cancer, and in vitro, in FRTL5 rat thyroid cells either chronically or acutely transformed by oncogenic H-Ras, where we invariably found that Klhl14 is downregulated compared to normal cells. Such reduction of Klhl14 in different experimental cancer models developed in different species further strengthens the original in silico observation. Analysis of Klhl14 promoter shows that its activity dramatically falls down in thyroid cells upon Ras oncogenic activation, suggesting that its transcriptional repression could represent an early event in thyroid neoplastic transformation. When Klhl14 was re-expressed in chronically transformed FRTL-5-HRas^V12^ cells, the number of obtained cell colonies was lower than the control and reduced proliferation was observed in both Klhl14-expressing mass cell population and individual clones. Notably, Klhl14-expressing pools and clones have a lower live-to-dead cell ratio, counted by exclusion methods. Consistently with this observation, growth curves of Klhl14-expressing cell pool and clones showed an apparent acceleration towards the plateau phase of growth and endpoint cell counts reduced between two- and four-fold compared to the respective controls. This agrees with the cell cycle analysis and propidium iodide uptake cell sorting experiments, where Klhl14-expressing cells persisted significantly more in G1 phase than control cells, suggesting a possible interference with the G1-S cell cycle checkpoint. The G1 phase delay in Klhl14 expressing cells is accompanied by a dramatic decrease in the number of duplicating cells counted by bromodeoxyuridine uptake. These alterations were even more evident when we consider the extent of DNA double-stranded breakage in our system. DNA laddering showed an increase in apoptotic cell death in Klhl14 expressing cells, especially when deprived of growth factors, namely in conditions mimicking the early phases of tumor growth, when neovascularization is still insufficient to ensure nutrient supply to neoplastic cells. Accordingly, Klhl14 expressing cells were more susceptible to apoptosis, as the rate of TUNEL positive cells arose more than six times compared to controls. Despite the sharp effects observed on cell proliferation and survival, Klhl14 exerts only mild effects on thyroid neoplastic cell differentiation, being able to reduce Tg protein expression, while not affecting its mRNA, and to further reduce NIS expression, both at the protein and the mRNA level. The continued expression of Klhl14 in thyroid follicular cells since the beginning of development prompted us to ask if it plays a role also in normally differentiated cells. Indeed, interfering Klhl14 expression reduced the protein abundance of the two transcription factors FoxE1 and Pax8 and of the iodine transporter NIS, all essential genes in thyroid function [16]. It is worth noting that FoxE1 and Pax8 protein decrease was not paralleled by a decrease in the respective mRNAs, which, on the contrary, significantly increase in level, suggesting an attempt of the follicular cell to replenish the decreased amounts of the encoded proteins to restore a fully differentiated phenotype. Another relevant aspect to consider is Klhl14 localization. Klhl14 has been originally detected in the ER and cytoplasm, consistently with its role of Cullin-RING Ligase 3 complex component [6], although recently it has been found expressed in the nucleus of malignant mesothelioma cells, and to shuttle between nucleus and cytoplasm in response to Tumor Growth Factor-β [7]. Accordingly, our data show that Klhl14 is localized in both the cytoplasm and nucleus of thyroid follicular cells, with a lower fraction in the nucleus of Ras-transformed cells compared to that of normal cells. We could hypothesize that Klhl14 may perform different functions in the two compartments, possibly playing multiple roles shaping the phenotype of neoplastic cells. Overall, our data highlighted for the first time the involvement of a ubiquitin ligase in thyroid cancer, where it appears to play a tumor suppressor-like role, by reducing proliferation and enhancing apoptosis. Further studies will be required to establish which of the observed effects are directly due to Klhl14 activity and which are instead mediated by its targets, and thus to identify the molecular network in which Klhl14 exerts its function.

## Materials and methods

### Differential expressed genes analysis

Two different gene expression datasets produced on platform GPL570 (Human Genome U133 Plus 2.0 Array, Affymetrix, Santa Clara, CA, USA), GSE65144 and GSE33630, were downloaded from Gene Expression Omnibus (http://www.ncbi.nlm.nih.gov/geo/). The GSE65144 contains 13 non tumoral and 11 ATC samples, whereas the GSE33630 contains 45 non tumoral, 12 ATC samples and 49 PTC samples. Differential Expressed Genes were analyzed and filtered using GEO2R tool (http://www.ncbi.nlm.nih.gov/geo/geo2r/). Genes with False Discovery Ratio adjusted *p* ≤ *0.05* and fold change (FC) ≥ 1 were considered being differentially expressed.

### Cell cultures and treatments

Normal non tumorigenic rat thyroid follicular cells FRTL-5, FRTL5-ER^TM^-Ras and tumorigenic clone FRTL-5-HRas^V12^ cells were grown in Coon’s modification of Ham’s F12 medium (ECM0019L/1, Euroclone, Pero, Italy) supplemented with 5% newborn calf serum (17682075, Cytiva HyClone, Fisher Scientific Italia, Segrate, Italy), penicillin/streptomycin, L-glutamine and a mix of six hormones containing 1 mU/mL TSH (Sigma-Aldrich Inc., Burlington, MA, United States), 10 mg/mL insulin (Sigma-Aldrich), 10 μg/mL somatostatin (Sigma-Aldrich), 5 mg/L transferrin (Sigma-Aldrich), 3.7 μg/L hydrocortisol, and 20 μg/mL Gly-His-Lys (Sigma-Aldrich) as described in De Vita et al. [19]. Cell lines were routinely tested for the presence of mycoplasma contamination by using EZ-PCR Mycoplasma Detection kit (Biological Industries, Beit-Haemek, Israel). Locked nucleic acid (LNA) oligonucleotides and plasmid DNA were transfected using Lipofectamine 2000® (Invitrogen, Life Technologies Ltd, Paisley, UK) according to manufacturer’s specifications. In total, 150 pmol of either targeting or scrambled LNA were used for each Klhl14 silencing experiments. Custom designed LNA were purchased from Qiagen (Qiagen GmbH, Hilden, Germany) with the following sequences: LNA targeting Klhl14: ATCGGCTGACAAAATT LNA scrambled control: AAGTGAGTGGAGGAGAGGA. FRTL-5-HRas^V12^ cells, a transformed FRTL5 clone that stably overexpress H-RAS^V12^ oncogene, was generated as a puromycin resistant clone after two-to-four weeks of selection, as described in De Vita et al. [19]. FRTL-5-HRas^V12^ Klhl14 cell line was developed from FRTL-5-HRas^V12^-V27 clone by stably transfecting full-length Klhl14 ORF and selecting using 400 μg/mL of G418 combined with supplemented media. Plasmids used for the generation of Klhl14 expressing FRTL-5-HRas^V12^-V27 cell pools and clones were 3xFlag-CMV10-Klhl14 containing the rat Klhl14 coding sequence flagged at N-terminal domain, and 3xFlag-CMV10 as empty vector control. FRTL-5-HRas^V12^-V27 cell pools and clones used in DNA fragmentation analyses were cultured for 72 h either in 5% NCS and standard amount of the mixture of six hormones or 0.1% NCS and absence of hormones.

### Mice and treatments

Double transgenic mice Tg-rtTA; TetO/BRAF^V600E^ were obtained by crossing Tg-rtTA and TetO/BRAF^V600E^ mice. Mice were maintained in the University of Naples “Federico II” (Naples, Italy) animal facility under pathogen-free condition, controlled temperature, humidity, and light cycle, and were supplied with standard or drug supplemented food and water *ad libitum* following the approval from Italian Ministry of Health (D.M. 78/213-A and 12/2018-UT) and from the Institutional Animal Care and Use Committee of the University of Naples “Federico II” with the protocol no. 2013/0078506. Tg-rtTA; TetO/*BRAF*^V600E^ mice were fed with 2,500 mg/kg doxycycline supplemented food for one week. According to the 3Rs principle to minimize the mouse sample number, we employed four (*N* = 4) mice either for the control and for the doxycycline-treated group, achieving statistically significant differences. No randomization and blinding criteria were applied. Mice used in this study all have the same genetic background (strain FVB/NJ).

### In situ hybridization

ISH was performed as described in [26]. Briefly, 7μm sections were obtained, deparaffinized and rehydrated from PFA 4%-fixed organs from adult mice. After rehydration, the hybridization was performed using digoxigenin-labeled riboprobes obtained using DIG-labeling RNA kit SP6/T7 (11175025910, Hoffmann-La Roche AG, Basel, Switzerland) following the manufacturer’s instructions. Probes were transcribed on previously amplified thyroid mouse cDNA using the following oligonucleotides: *Klhl14 F:* GAGGATACAGCTGGAGTATGGG, *Klhl14 R*: GAGCTGAAGAGCAATAGGGTGT. Micrographs were obtained using Axioskop microscope and acquisitions were made with Axiocam 105 color digital camera (Carl Zeiss AG, Oberkochen, BW, Germany) and ultimately processed with built-in Axion Vision software.

### Luciferase reporter gene analysis

The reporter containing plasmid was generated cloning the 0.5 kb region upstream the first ATG of *Klhl14* mouse gene, obtained using the following oligonucleotides: *0.5kb_Klhl14 F:* CGCGACGCGTCTTCCAGCACCAGTTGACCT, *0.5kb_Klhl14 R:* GGAAGATCTCTGACTCTGCGCACCTGG. Restriction enzymes BglII (New England Biolabs, Ipswich, MA, United States) and MluI (New England Biolabs) were used to introduce the gel purified amplification product in promoterless firefly luciferase reporter vector pGL3Basic (Promega Corporation, Madison, WI, United States) that was previously linearized with the same enzymes, dephosphorylated with calf intestinal alkaline phosphatase (M1821, Promega Corporation, Madison, WI, United States) and gel purified. Luminescence signals from samples were normalized using signal intensities from the internal control *Renilla* luciferase expressed via co-transfection of pRL-TK control reporter vector (Promega Corporation).

### RNA isolation and quantitative Real-time PCR

Total RNA was isolated from cultured cells using Trizol® reagent (Life Technologies, Grand Island, NY, United States) according to manufacturer’s specifications. Total cDNA was generated with SuperScript III Reverse Transcriptase (Thermo Fisher Scientific, Waltham, MA, United States) according to manufacturer’s specifications. Real-Time PCR on total cDNA was performed with Universal SYBR Green supermix (Bio-Rad, Hercule, CA, United States) using gene specific oligonucleotides purchased from Eurofins Scientific SE (Luxembourg City, Luxembourg): PanrKlhl14 F: CGATGACAGCATTTATCTAGTTGG, PanrKlhl14 R: GCAGATTGTAGAGGACTTGTAGGC, rThybe1 F: GGCTCCTCTCCACTCACTTTC, rThybe1 R: TCAGCTCAGCAGCGAAGTC, rPax8 F: GCCATGGCTGTGTAAGCAAGA, rPax8 R: GCTTGGAGCCCCCTATCACT, rFoxE1 F: AAGCCGCCCTACAGCTACATCG, rFoxE1 R: AACATGTCCTCGGCGTTGGG, rNis F: TCCACAGGAATCATCTGCACC, rNis R: CCACGGCCTTCATACCACC, rThyroglobulin F: CATGGAATCTAATGCCAAGAACTG, rThyroglobulin R: TCCCTGTGAGCTTTTGGAATG, 18 S F: CGGCTACCACATCCAAGGAA, 18 S R: GGGCCTCGAAAGAGTCCTGT. Each reaction contained 10 µL of 2× Sybr Green, 200 nM of each primer, and 20 ng of cDNA.

### Protein extraction, Nucleus/cytoplasm fractionation and western blot

Whole cell lysates were obtained using a lysis buffer made of NaCl 150 mM, Tris HCl 50 mM, MgCl2 5 mM, sodium deoxycolate 0.5%, sodium dodecyl sulfate (SDS) 0.1%, Triton X-100 1%. To this buffer were added Dithiothreitol (Sigma-Aldrich) 0.1 mM, Phenylmethylsulfonate fluoride (Sigma-Aldrich) 0.5 mM, proteinase inhibitor cocktail (P8340, Sigma-Aldrich) and phosphatase inhibitor cocktail (P0044, Sigma-Aldrich). Cell suspensions were incubated 30 min on shaker at +4 °C and the derived lysate were cleared by centrifugation at 20817 g for 20 min at +4 °C. PARIS^TM^ kit (AM1921, Invitrogen, Waltham, MA, United States) was used for Nucleus/Cytoplasm fractionation following the manufacturer’s instructions. Protein concentrations were measured via BCA assay and 20 μg or 50 μg were loaded for western blot. Immunoblot were incubated with the following antibodies: rabbit polyclonal antibodies against FoxE1, Pax8 and Nis, previously produced in our laboratory were used at approximately 1 µg/mL [11, 20], anti-Bcl2 (sc-7382, Santa Cruz Biotechnology, Dallas, TX, United States), anti-Thyroglobulin (M0781, Agilent Technologies Inc., Santa Clara, CA, United States), anti-Gapdh (sc-32233, Santa Cruz Biotechnology), anti-Klhl14 (MBS2526408, MyBioSource Inc., San Diego, CA, United States), anti-H3 (ab1791, Abcam Lt., Cambridge, United Kingdom). Experiments conducted in FRTL5 parental cells required 20 µg of whole cell lysate loaded on NuPAGE^TM^ 4–12%, Bis-Tris Mini Protein gels, while experiments in FRTL-5-RAS clones required 50 µg of whole cell lysate. Experiments conducted on Nucleus/Cytoplasm fractionates were carried out by loading 10 μg of each fractionate on Bolt^TM^ Bis-Tris Mini Protein Gels, 4–12% 1.0 mm (NW04125BOX, Invitrogen). Full and uncropped western blots are available as a supplementary file of the online version.

### Viable cell counting, MTS assay and clonogenic assay

Cell viability was assessed via Trypan Blue exclusion assay using 0.4% Trypan Blue solution and counting cells with Bürker counting chamber. MTS colorimetric assay was performed using CellTiter 96® AQueus One Solution Cell Proliferation Assay (G3582, Promega Corporation, Madison, WI, United States). Briefly, selected RAS-V12 FRTL-5 Klhl14 cells and their Empty Vector control were counted and plated at the same density on day 0 and let outgrown for 48 h; after 48 h they were replated in a 96-well plate, incubated for two hours with the MTS solution, and scanned using BioTek Synergy HT microplate reader (BioTek Instruments, Inc., Winooski, VT, United States). Clonogenic assay was performed as previously described [12]; after approximately three weeks of selection, cells passaged at two different split ratios (1:6 and 1:10) were treated with a 6% Glutaraldehyde + 0.4% Crystal Violet solution for 30 min at room temperature and then the excess was washed pipetting water into the dishes.

### Flow cytometry and Propidium Iodide analyses

Propidium Iodide cell cycle and inclusion analyses were performed as in [27]. Briefly, cells passaged 48H before the experiment were harvested and permeabilized using 100% ethanol for cell cycle and kept an overnight at −20 °C. After this, cell suspension was spun, washed with PBS and resuspended at the concentration of 2 × 10^3^ cells/µL in Propidium Iodide staining solution (50 µg/mL PI, 100 mg/mL RNase A, 1% NP-40) and analyzed with FACS Calibur cytofluorimeter (BD Biosciences, Franklin Lakes, NJ, United States). For Propidium Iodide inclusion assay, cells were treated with the same staining protocol, except for permeabilization steps and without employing NP-40 in staining solution. For each sample, 10^4^ events were recorded and raw data were analyzed using CellQuest software (BD Biosciences).

### DNA fragmentation analysis

Cells (1 × 10^6^ per sample) were lysed in 0.5% Triton X-100, 5 mM Tris Buffer (pH 7.4), and 20 mM EDTA for 20 min at 4 °C. After centrifugation at 14,000 rpm in a microcentrifuge, supernatants were extracted with phenol-chloroform and precipitated in ethanol. Soluble DNA was incubated with 50 μg/mL RNase A for 1 h and electrophoresed on a 1.2% agarose gel.

### S-phase entry-BrdU incorporation and TUNEL assays

BrdU proliferation assay were carried as manufacturer’s specifications for 5-Bromo-2’-deoxyuridine Labeling and Detection Kit (11296736001, Merck KGaA., Darmstadt, Germany). Briefly, cells were plated and grown until they reached 50% confluency; then they were incubated with BrdU labeling solution at 1H at 37 °C and 5% pCO_2_: positive cells were assayed using Zeiss LSM700 confocal microscope. Terminal deoxynucleotidyl transferase dUTP nick end labeling (TUNEL) assay were performed as manufacturer’s specification using the In Situ Cell Death Detection Kit, Fluorescein kit (11684795910, Hoffmann-La Roche). Cells were maintained at a confluency of around 80% on 12 mm diameter culture-suitable coverslips and fixed as per procedure. After permeabilization, they were incubated 1H at 37 °C with labeling buffer containing TdT polymerase, in a humidified atmosphere in the dark. The buffer was then removed and coverslips were washed with PBS. Cells from both experiments, were analyses under LSM700 laser scanning confocal microscope (Carl Zeiss AG, Oberkochen, BW, Germany).

### Immunofluorescence

Cells were plated 1.2 ⨯ 10^4^ cells/cm^2^ on 12 mm sterilized uncoated glass coverslips no. 1.5 thickness (Ibidi GmbH, Gräfelfing, BY, Germany), that have been previously placed in a 60 mm cell culture dish and grown until 50% confluent (which approximately took 48 h from seeding). Cells were then briefly washed three times with 1X DPBS, fixed with 4% PFA in PBS for 15 min. Free aldehydes were quenched with 50 mM NH_4_Cl and the cells were washed three times with 1X PBS. Cells were then permeabilized with 0.1% Triton X-100 in PBS for 10 min, washed again three times and finally blocked with 5% BSA in PBS for 30 min. Subsequent incubations were made using primary antibody anti-Klhl14 (MBS2526408) 1:100 incubated over-night at 4 °C, followed by secondary antibody AlexaFluor^TM^ Plus 555 Goat anti-Rabbit IgG (A32732, Invitrogen) 1:400 incubated at room temperature for 1 h. Nuclear counterstaining was achieved using Hoechst 33342, diluted 1:2000 in a 1:1 Glycerol:PBS mounting medium. Cells were analyzed under LSM700 laser scanning confocal microscope (Carl Zeiss AG).

### Statistical analysis and software

Statistical analyses were performed at least on three independent biological replicates, unless otherwise stated. Sample size was chosen based on preliminary experiments conducted on FRTL5 and derived FRTL-5-HRas^V12^ aimed at calculating effect size and estimating the associated statistical power. Homoscedastic, two-tailed t-Student’s test were performed on qRT-PCR and immunoblot densitometries, assuming a normal distribution and similar variances a priori. In each experiment, the significance was evaluated at *p* < *0.05*. In each figure where it applies, the mean values and standard deviation (SD) of the data are presented. All acquisitions and micrographs, as well as immunoblot films were analyzed using FiJi software (Image J, National Institutes of Health, Bethesda, MD, United States). Immunofluorescence micrographs were analyzed using ZEN 3.8 (Carl Zeiss AG, Oberkochen, BW, Germany) and using JACoP FiJi plugin software for co-localization measurements [28]. Pearson’s Colocalization Coefficient (r_p_) and Manders’ Overlap Coefficients (MOCs) were analyzed using the Costes method for estimating threshold.

### Supplementary information

Supplemental Material - Original Blots

## Data Availability

All the data produced with this study, together with the original exposed films of each immunoblot presented, are included in this published article and its supplementary information files. Validating datasets referred in this study are free and publicly available as stated in their respective source article indicated along the manuscript.
