# Supplementary material for: KLHL14 is a tumor suppressor downregulated in undifferentiated thyroid cancer
Source: Cell Death Discov. 2024 Jun 22;10:297. doi: 10.1038/s41420-024-02063-7 (PMC11193815; doi:10.1038/s41420-024-02063-7)
Supplement: Supplementary file 1 — Supplemental Material - Original Blots [file 41420_2024_2063_MOESM1_ESM.pdf]

A

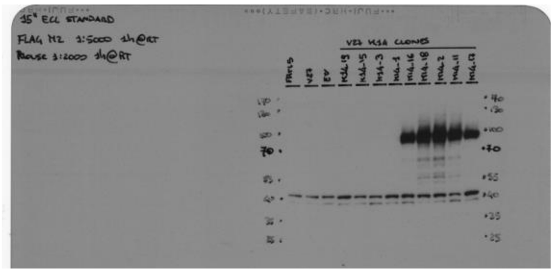

B

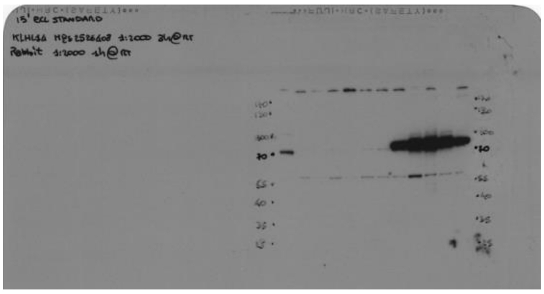

C

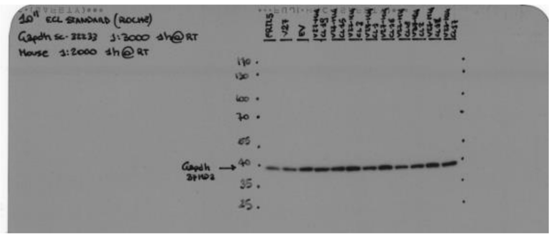

Supplementary Figure 1: Uncropped blots presented in Fig.2C.

A

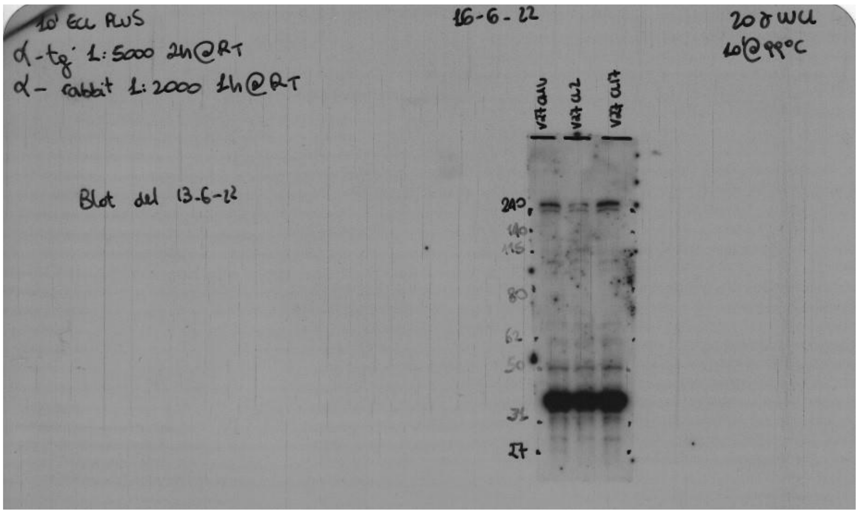

B

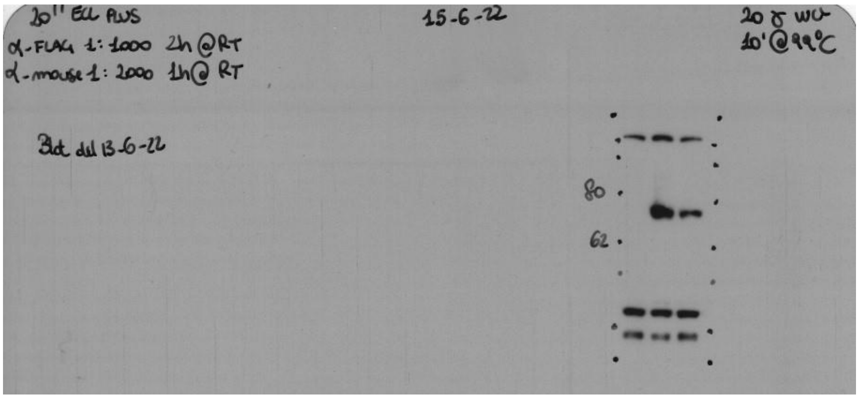

C

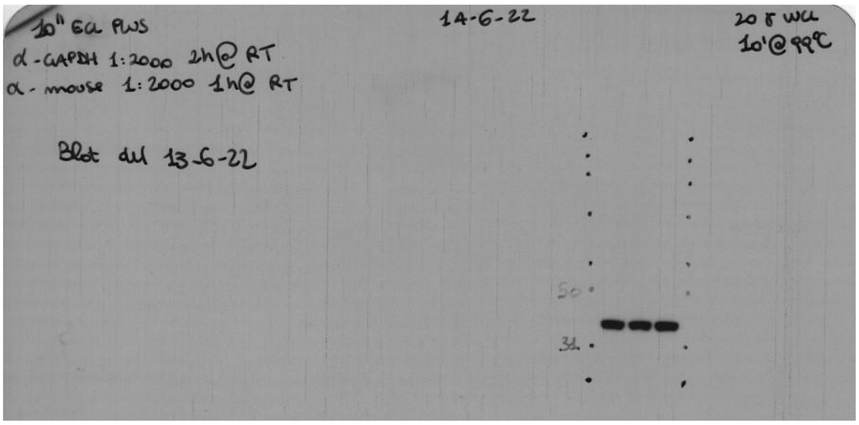

Supplementary Figure 2: Uncropped blots presented in Fig.5A.

A

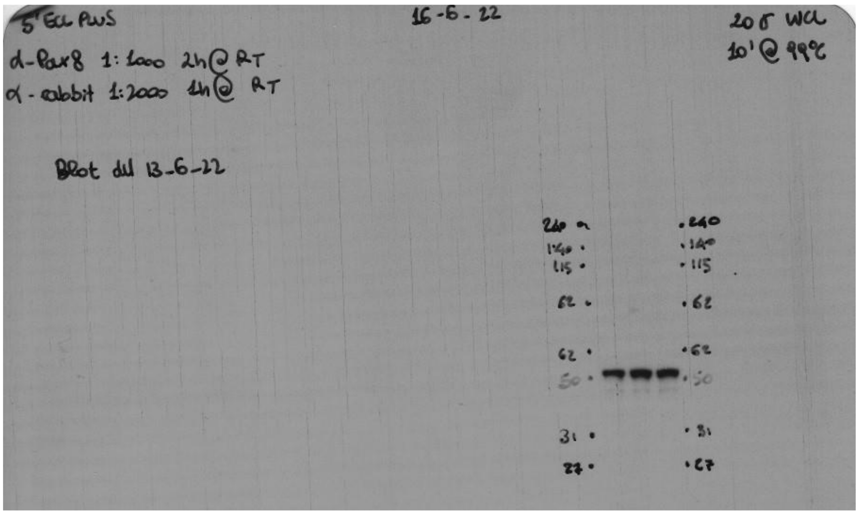

B

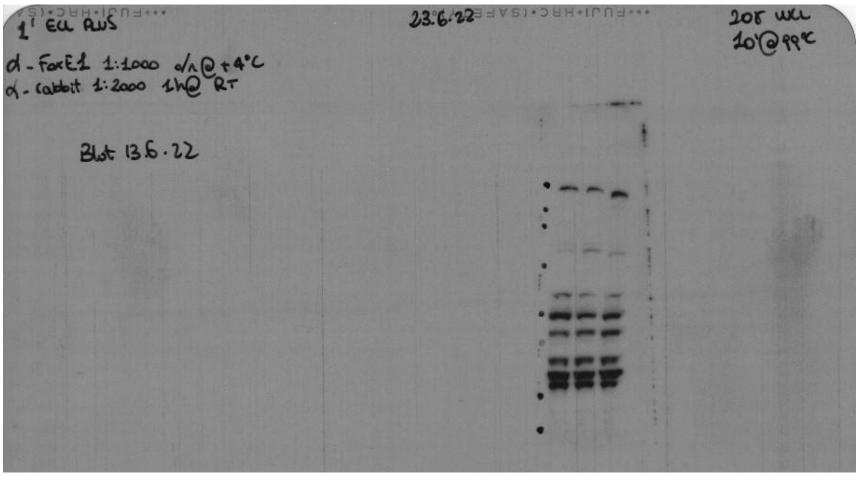

C

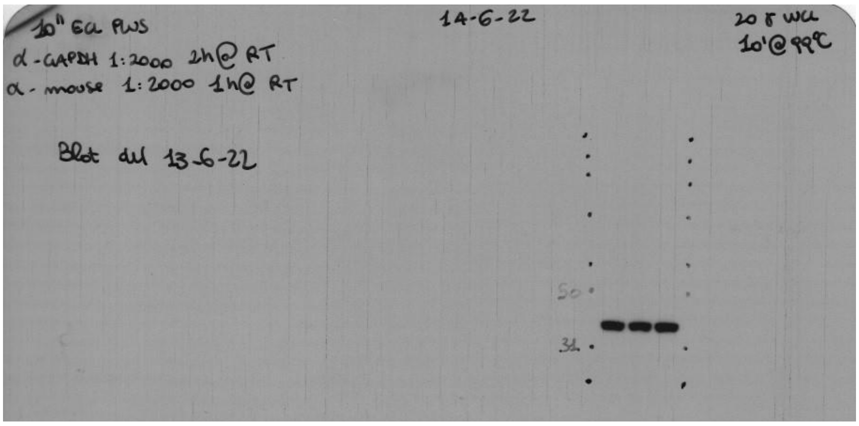

Supplementary Figure 3: Uncropped blots presented in Fig.5A.

A

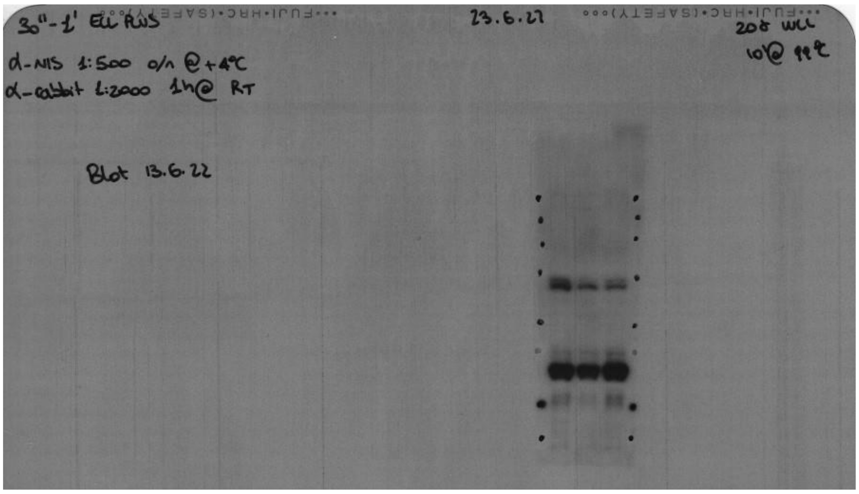

B

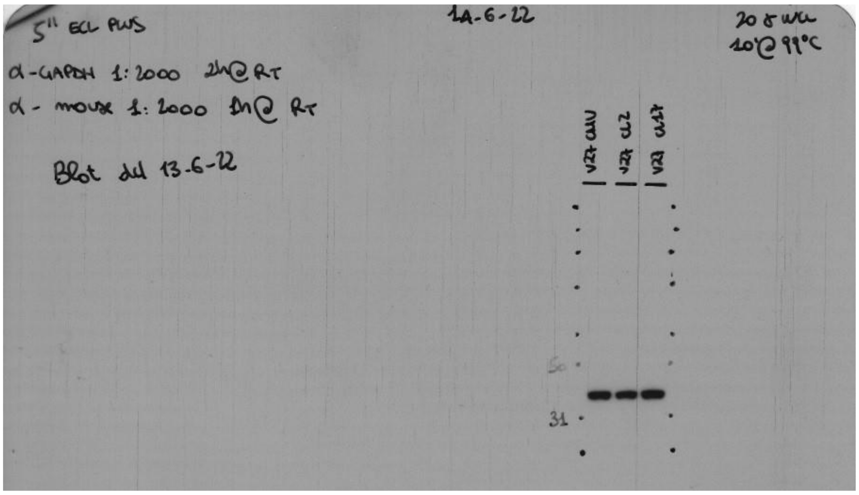

Supplementary Figure 4: Uncropped blots presented in Fig.5A.

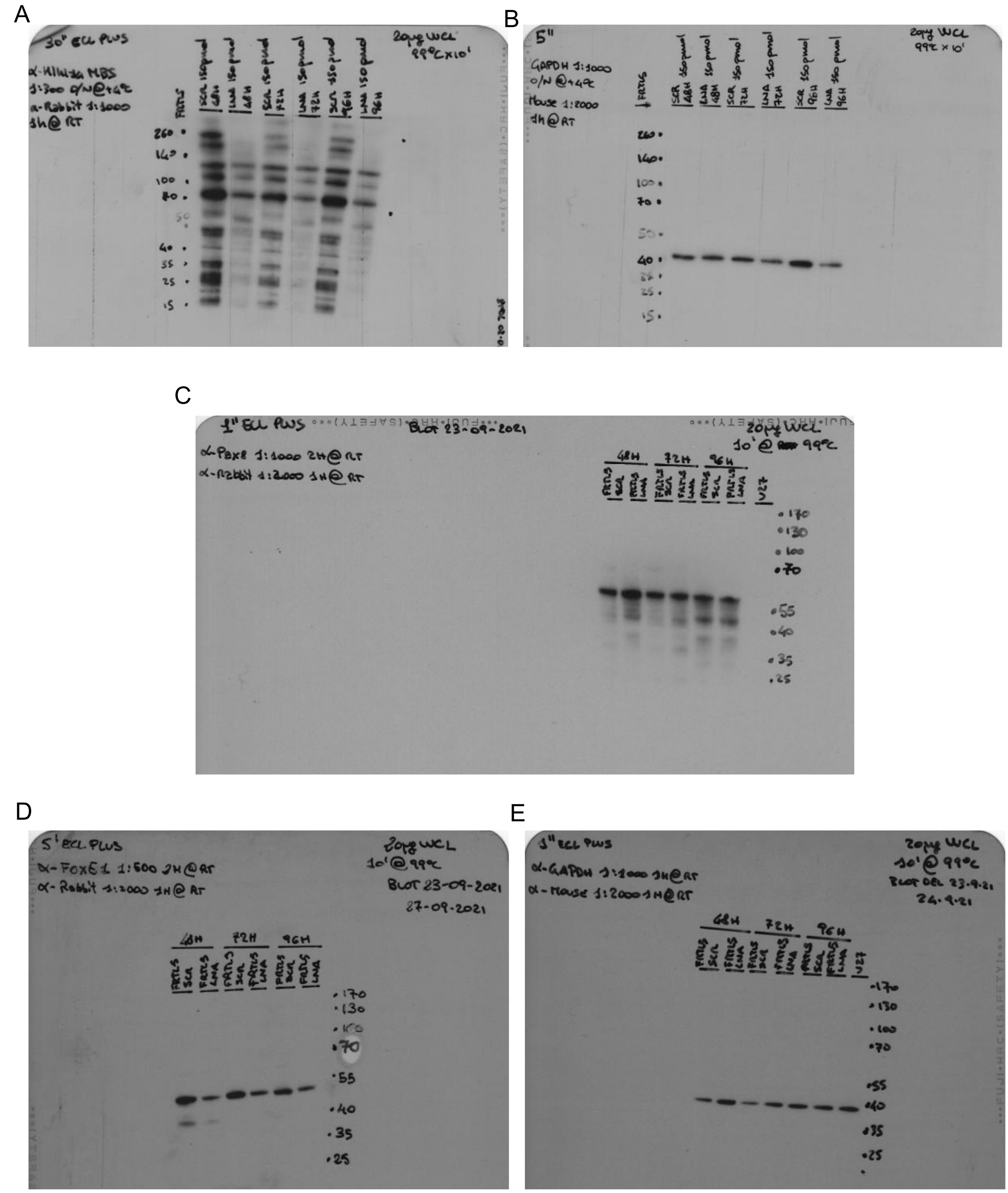

Supplementary Figure 5: Uncropped blots presented in Fig.6A.

A

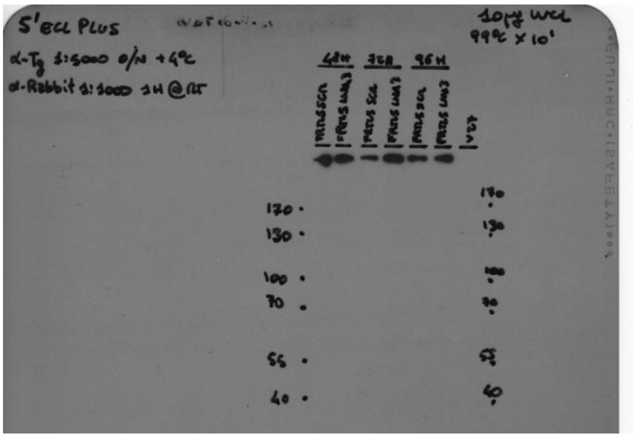

B

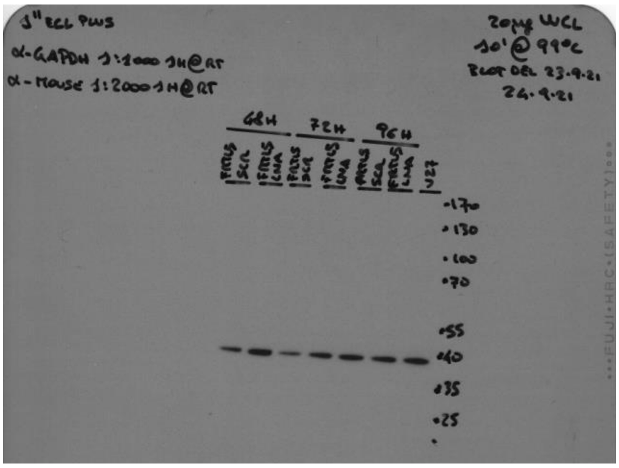

Supplementary Figure 6: Uncropped blots presented in Fig.6A.

A

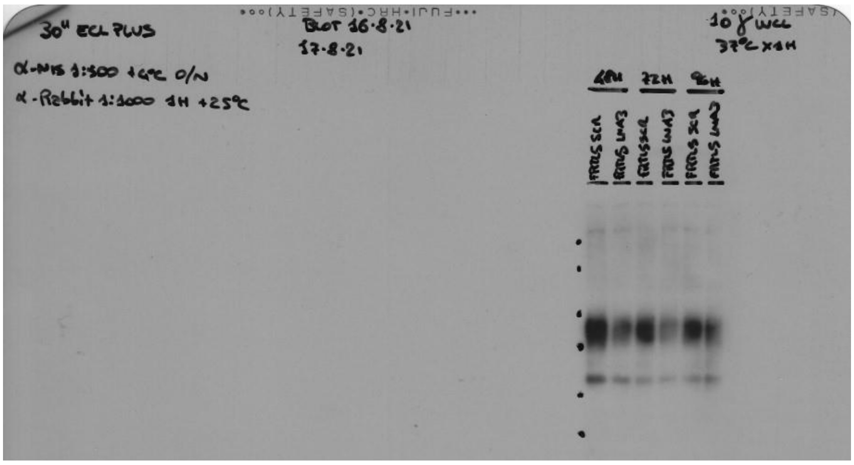

B

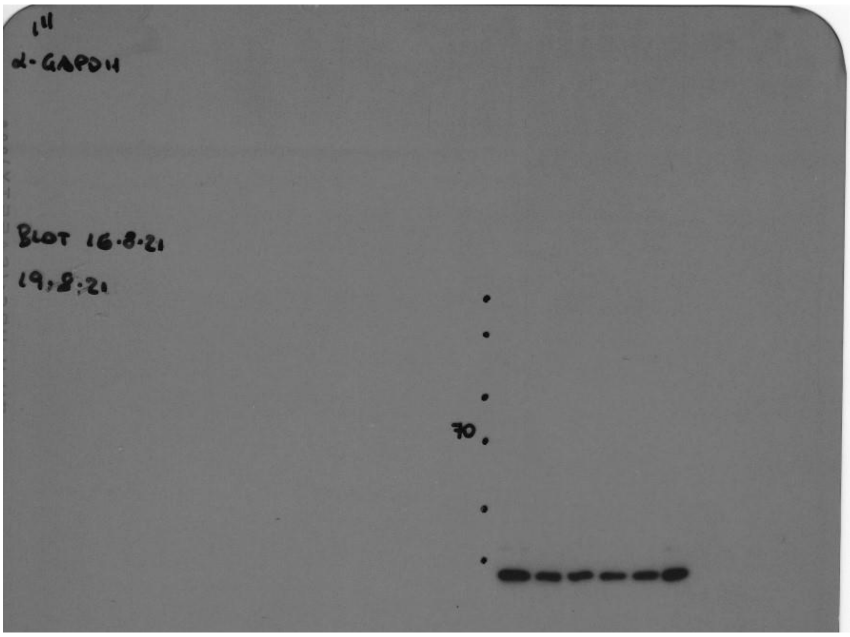

Supplementary Figure 7: Uncropped blots presented in Fig.6A.

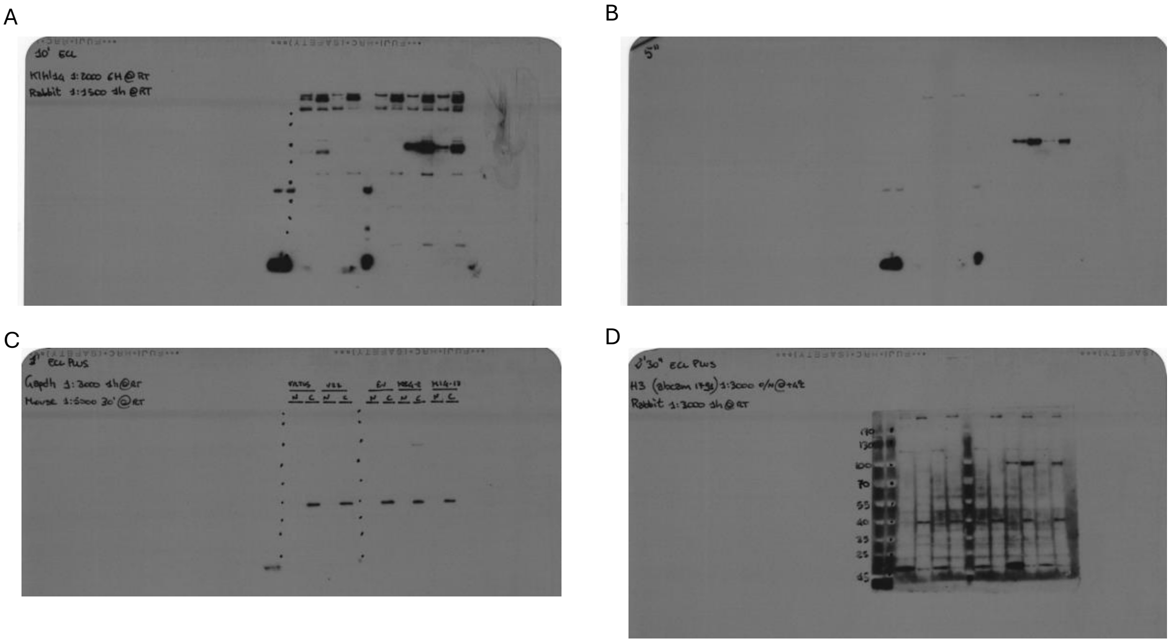

Supplementary Figure 8: Uncropped blots presented in Fig.7A.
